# Neural-Network-Based Model-Free Calibration Method for Stereo Fisheye Camera

**DOI:** 10.3389/fbioe.2022.955233

**Published:** 2022-07-14

**Authors:** Yuwei Cao, Hui Wang, Han Zhao, Xu Yang

**Affiliations:** ^1^ School of Automation, Wuhan University of Technology, Wuhan, China; ^2^ Key Laboratory of Icing and Anti/De-icing, China Aerodynamics Research and Development Center, Mianyang, China; ^3^ School of Integrated Chinese and Western Medicine, Anhui University of Chinese Medicine, Hefei, China

**Keywords:** fisheye camera, stereo calibration, phase unwrapping, neural-network, large field of view

## Abstract

The fisheye camera has a field of view (FOV) of over 180°, which has advantages in the fields of medicine and precision measurement. Ordinary pinhole models have difficulty in fitting the severe barrel distortion of the fisheye camera. Therefore, it is necessary to apply a nonlinear geometric model to model this distortion in measurement applications, while the process is computationally complex. To solve the problem, this paper proposes a model-free stereo calibration method for binocular fisheye camera based on neural-network. The neural-network can implicitly describe the nonlinear mapping relationship between image and spatial coordinates in the scene. We use a feature extraction method based on three-step phase-shift method. Compared with the conventional stereo calibration of fisheye cameras, our method does not require image correction and matching. The spatial coordinates of the points in the common field of view of binocular fisheye camera can all be calculated by the generalized fitting capability of the neural-network. Our method preserves the advantage of the broad field of view of the fisheye camera. The experimental results show that our method is more suitable for fisheye cameras with significant distortion.

## 1 Introduction

The ordinary camera has a limited FOV. It can no longer meet the needs of some research projects without adding other auxiliary facilities. The appearance of the fisheye camera overcomes this shortcoming. The fisheye camera has a small focal length, and the field of view can generally reach more than 180° (Arfaoui and Thibault, 2013). Due to the large field of view of the fisheye camera, one single fisheye image can show a large portion of the surrounding environment without image splicing (Hou et al., 2012).

The research on stereo calibration technology of fisheye cameras is more meaningful. Compared with ordinary camera, the structure of fisheye camera is more complicated. Fisheye cameras introduce severe distortion, especially barrel distortion, during imaging (Kanatani, 2013). This strong optical distortion results in high image center separation and low resolution at the edges of fisheye images (Hughes et al., 2010). Consequently, the stereo calibration accuracy of the fisheye camera is also limited to some extent.

The traditional stereo calibration method for fisheye cameras requires an imaging model in a specific mathematical format. Before this paper, there were some studies on stereo calibration techniques for fisheye cameras. For example, first combined fisheye camera calibration and epipolar rectification applied in a stereo fisheye camera system. They accomplished 3D reconstruction of specific points from authentic fisheye images Abraham and Forstner (2005). Designed a novel measurement system based on a binocular fisheye camera. The measurement system uses the dynamic angle compensation method, which can achieve high-precision 3D positioning in a dynamic environment Cai et al. (2019). Proposed a new strategy for computing parallax maps from hemispherical stereo images taken by fisheye camera. They considered only matches of the same class by segmenting the textures in the scene Herrera et al. (2011). Presented a method to calibrate multiple fisheye cameras with a wand that can move freely. The internal and external parameters and the 3D coordinates of the fisheye camera could be obtained Fu et al. (2015). Proposed a panoramic stereoscopic imaging system, which could provide stereoscopic vision of 360° horizontal field Li and Li (2011). Analyzed existing dense stereo systems. They combined the epipolar rectification model of the binocular fisheye camera with the dense method, able to provide dense 3D point clouds at 6–7 Hz Schneider et al. (2016). These stereo calibration methods usually require correcting fisheye images with significant distortion to perspective projection images. However, this distortion removal process leads to the loss of information at the image edges, losing the advantage of the large field of view of the fisheye camera. So the results of performing stereo matching on fisheye images are unsatisfactory. In addition, stereo matching also has strict restrictions on the scene. Some factors such as too much scene noise pollution and too much repetitive texture may impact the matching accuracy.

With the development of artificial intelligence, Deep Learning (DL) is increasingly used in the field of computer vision (Huang et al., 2021). There have been many studies applying DL to the distortion correction of fisheye images. Proposed a Distortion Rectification Generative Adversarial Network (DR-GAN) for the severe barrel distortion of wide-angle camera images. DR-GAN is the first end-to-end trainable adversarial framework for radial distortion correction Liao et al. (2020). Considered the characteristics of fisheye images and proposed an unsupervised fisheye camera distortion correction network. The network can predict distortion parameters and implement direct mapping from fisheye images to rectified images Yang et al. (2020). DL-based methods are computationally fast. However, they trained the network with a large number of fisheye images, which consumes a lot of resources. In addition, this method is very sensitive to the scene.

To overcome these shortcomings, we propose the application of neural-network to the stereo calibration of binocular fisheye camera. Take the image coordinates of the left and right fisheye cameras as the input training set. The spatial coordinates corresponding to the image coordinates in the scene are used as the output training set. The trained neural-network can implicitly describe the mapping relationship from the 2D image plane to the 3D space. With the nonlinear fitting ability of the neural-network, it can directly predict the spatial coordinates of the target point based on the trained network. Compared with traditional stereo calibration, the proposed method is model-free. There is no need to establish an accurate mathematical imaging model, nor does it need to know the intrinsic and extrinsic parameters of the fisheye camera. Experiments have been conducted, and their results verify the performance of the proposed method.

To obtain the training set of the neural-network, a large number of feature points with known image coordinates and spatial coordinates are required. Some 2D targets such as chessboard are the most commonly used. The chessboard-based calibration method has good calibration accuracy for ordinary cameras (Zhang, 2000). However, the chessboard images taken by the fisheye camera have severe barrel distortion, which leads to low feature detection accuracy or failure to detect feature points located at the edge of the images. To overcome this shortage, active targets are used (Schmalz et al., 2011). Active phase targets are widely used in optical measurement due to their high accuracy and high speed (Wang et al., 2011; Xu et al., 2017; Wang et al., 2020; Wang et al., 2022). This paper uses a feature extraction method based on three-step phase-shift method and a multi-frequency method (Wang et al., 2019). This feature extraction method has high precision and strong robustness (Schmalz et al., 2011). Therefore, it is more suitable for fisheye cameras with severe distortion.

The remained parts of the paper are as follows. Section 2 describes the fisheye camera model and the stereo calibration model of the binocular fisheye camera. Section 3 presents the training process of the neural-network, the setting of the neural-network parameters, and the acquisition of the training sets. Section 4 describes the experiment. Finally, Section 5 concludes as well as some prospects for the future.

## 2 Principle

### 2.1 Single Fisheye Camera Model

Fisheye cameras take non-similar imaging and introduce large barrel distortion in the imaging process. Compressing the diameter space breaks the limitation of the imaging field of view to achieve wide-angle imaging (Wei et al., 2012). The projection refraction angle and the incident angle of fisheye cameras are not equal and will deviate from the direction of the optical axis for refraction. There are four basic imaging models of fisheye cameras: equidistant projection model, equisolid-angle projection model, orthographic projection model, and stereographic projection model (Schneider et al., 2009).

The projection equation for equidistant projection is shown as: Eq. 1

$$\begin{array}{c} r_{d}=f\theta \end{array}$$ (1)

where $r_{d}$ is the radial distance; f is the focal length of the fisheye camera; θ is the angle of incidence of the light.

The projection equation for equisolid-angle projection is shown as: Eq. 2

$$\begin{array}{c} r_{d}=2fsin\left(\frac{\theta }{2}\right) \end{array}$$ (2)

The projection equation for orthographic projection is shown as: Eq. 3

$$\begin{array}{c} r_{d}=fsin\theta \end{array}$$ (3)

The projection equation for stereographic projection is shown as: Eq. 4

$$\begin{array}{c} r_{d}=2ftan\left(\frac{\theta }{2}\right) \end{array}$$ (4)

Traditional distortion models cannot guarantee the accuracy of fisheye camera. Several models have been developed to represent the distortion of the fisheye camera, including the polynomial model, the field-of-view (FOV) model (Devernay and Faugeras, 2001), and the fisheye transform (Base, 1995).

### 2.2 Stereo Calibration Model of the Binocular Fisheye Camera

**FIGURE 1:**
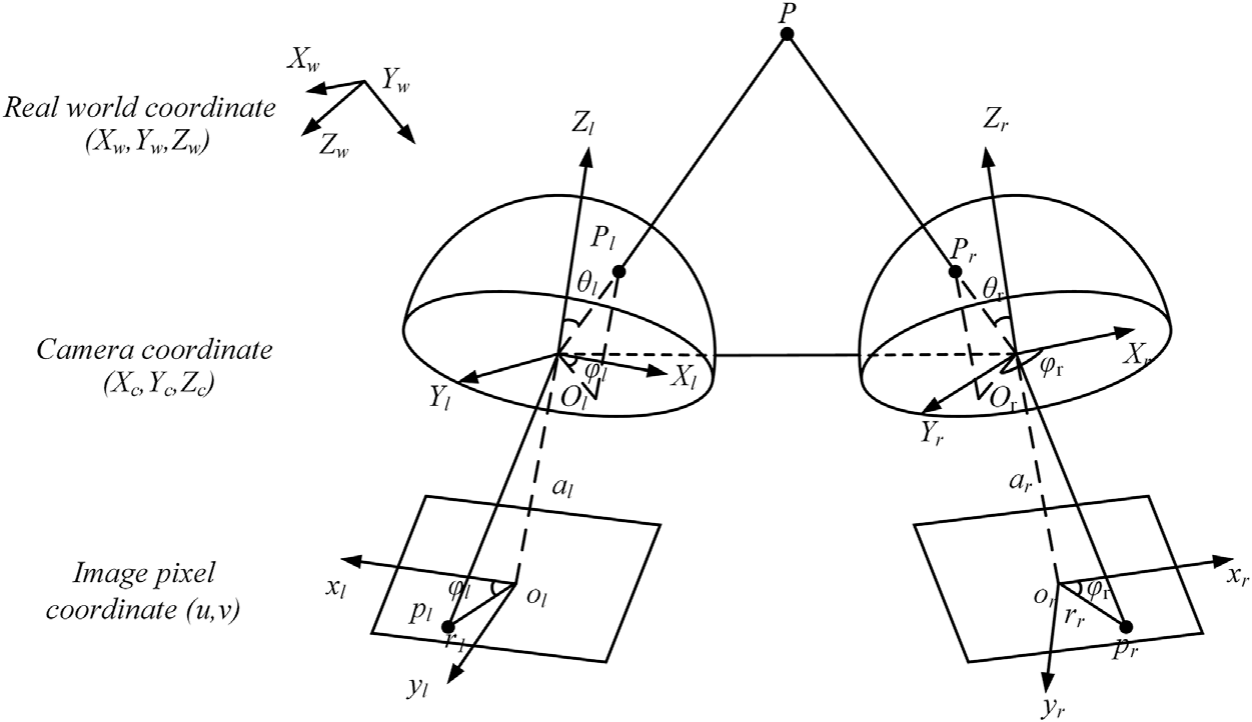
Stereo calibration model of the binocular fisheye camera.

Figure 1 shows the stereo calibration model of the binocular fisheye camera. The fisheye camera coordinate system on the left is denoted by $O_{l}-X_{l}Y_{l}Z_{l}$ , and the fisheye camera coordinate system on the right is denoted by $O_{r}-X_{r}Y_{r}Z_{r}$ . Since the fisheye camera imaging is nonlinear, the camera coordinate system is denoted by unit spherical coordinates. The world coordinate system is denoted by $O_{w}-X_{w}Y_{w}Z_{w}$ . The relative positions of the left and right cameras are fixed, and their relationship can be expressed as:

$$\begin{array}{c} P_{l}=R_{l}P_{w}+T_{l} \end{array}$$ (5)

$$\begin{array}{c} P_{r}=R_{r}P_{w}+T_{r} \end{array}$$ (6)

where $R_{l}$ and $R_{r}$ represent the rotation vectors corresponding to the world coordinate system and the left and right fisheye camera coordinate systems, respectively; $T_{l}$ and $T_{r}$ represent the translation vectors; $P_{w}$ represents the world coordinate of any point P ; $P_{l}$ and $P_{r}$ respectively represent the coordinate of the point P in the left and right camera coordinates. Combining Eqs 5, 6, we can obtain the spatial position conversion relationship between the left and right camera coordinate systems: Eq. 7

$$\begin{array}{c} P_{r}=R_{r}R_{l}^{-1}P_{l}+T_{r}-R_{r}R_{l}^{-1}T_{l} \end{array}$$ (7)

Therefore, the rotation vector of the left fisheye camera to the right fisheye camera is $R=R_{r}R_{l}^{-1}$ , and the translation vector is $T=T_{r}-R_{r}R_{l}^{-1}T_{l}$ .

The solution R of and T is essentially the process of stereo calibration (Li, 2008; Beekmans et al., 2016).

## 3 Neural-Network Model

### 3.1 Training Process of Neural-Network

**FIGURE 2:**
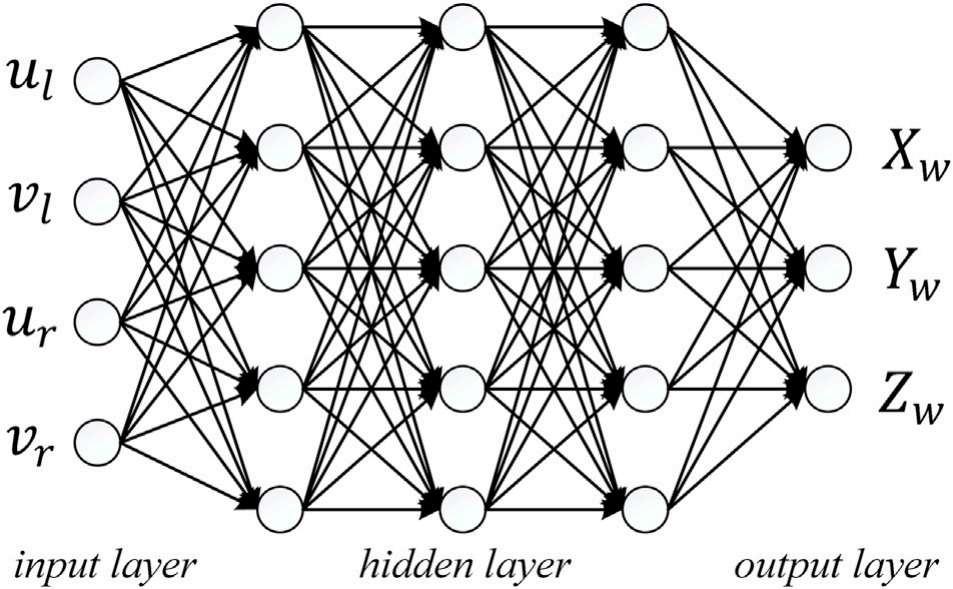
Structure of the neural-network.

In recent years, the emergence of some bio-inspired algorithms that simulate natural ecosystems provide new ideas for solving complex optimization problems. These bio-inspired algorithms include genetic algorithms (Liu X et al., 2022), particle swarm algorithms (Liu Y et al., 2022; Wu et al., 2022; Zhao et al., 2022), predictive modeling algorithms (Chen et al., 2021a; Chen et al., 2021b; Chen et al., 2022), convolution neural network algorithm (Huang et al., 2022; Tao et al., 2022; Yun et al., 2022), artificial bee colony algorithm (Sun et al., 2022), etc. A neural-network is a multi-layer feed forward network that follows an error back propagation algorithm, as shown in Figure 2. The basic component units of a neural-network are neurons, also called network nodes. The essence of each neuron is a nonlinear transformation of the input data. Theoretically, a neural-network can accomplish any form of nonlinear mapping (Parma et al., 1999). A neural-network can provide a nonlinear model $h_{w,b}\left(x\right)$ to fit the output y . The network parameters are the weights w and the bias b . Training a neural-network is to continuously update these two parameters under the stimulus of external inputs so that the output keeps approaching the desired output. The training process consists of a forward propagation process of the input information and a backward propagation process of the error information.

The forward propagation process can be understood as follows: the output of the previous layer is used as the input of the next layer, and the output of the next layer is calculated until the operation reaches the output layer. Let the activation value of the $i^{th}$ node in the $l^{th}$ layer of the neural-network be $a_{i}^{l}$ . The $j^{th}$ node in the ${\left(l-1\right)}^{th}$ layer to the $i^{th}$ node in layer $l^{th}$ node is connected by the weight $w_{ij}^{l}$ . $b_{i}^{l}$ is the bias of the $i^{th}$ node in layer $l^{th}$ . It is not difficult to see from the structure of the neural-network that $a_{i}^{l}$ depends on the activation of the previous layer.

$$\begin{array}{c} a_{i}^{l}=f\left(\sum w_{ij}^{l}a_{i}^{l-1}+b_{i}^{l}\right) \end{array}$$ (8)

where f() is the activation function. In this paper, the activation function uses the tanh function. Expressing Eq. 8 in matrix form:

$$\begin{array}{c} \{\begin{array}{c} z^{l}=W^{l}a^{l-1}+b^{l}\\ a_{i}^{l}=f\left(z^{l}\right) \end{array} \end{array}$$ (9)

where $z^{l}$ is the input of each layer. Use Eq. 9 to calculate the activation value of the network layer by layer. Finally, the output of the network can be obtained.

Before explaining the back propagation algorithm, it is first necessary to define the loss function. The loss function can measure the loss between the output computed by the training samples and the actual output.

The purpose of the back propagation process is to adjust the network parameters. Its essence is to find the optimal weights and biases by minimizing the loss function. So it is necessary to calculate the partial derivatives of the loss function to the weights and biases. The gradient of the variables in each layer of the neural-network can be obtained by finding the partial derivatives. The stochastic gradient descent algorithm (SGD) is commonly used to update the network parameters. The SGD algorithm can be summarized as: Eq. 10

$$\begin{array}{c} \{\begin{array}{c} w_{ij}^{l}=w_{ij}^{l}-\eta \frac{\partial }{\partial w_{ij}^{l}}J\left(\right)\\ b_{i}^{l}=b_{i}^{l}-\eta \frac{\partial }{\partial b_{i}^{l}}J\left(\right) \end{array} \end{array}$$ (10)

where η is the learning rate; J() is the loss function. After the network parameters are updated, we determine whether the current model meets the requirements. If the requirements are not met, the following forward and backward propagation is performed. The network parameters continue to be updated. Until the current model meets the requirements, the neural-network training is completed.

### 3.2 Setting of Neural-Network Parameters

This paper uses a neural-network to implicitly describe the nonlinear mapping relationship between image coordinates and their corresponding spatial coordinates in the scene. The settings of neural-network parameters include the structure of the neural-network, loss function, activation function, and optimizer. With the training sets keep constant, different network parameters can significantly impact the convergence speed and prediction accuracy of the network.

#### 3.2.1 Structural Parameters

The neural-network structure proposed in this paper contains five layers, as shown in Figure 2. There is one input layer, three hidden layers, and one output layer. The input layer has four nodes. $\left(u_{l},v_{l}\right)$ represent the left image coordinates of feature points. $\left(u_{r},v_{r}\right)$ represent the right image coordinates of feature points. The output layer has three nodes. $\left(X_{w},Y_{w},Z_{w}\right)$ represents spatial coordinates of feature points. Each hidden layer contains five nodes.

#### 3.2.2 Loss Function

**FIGURE 3:**
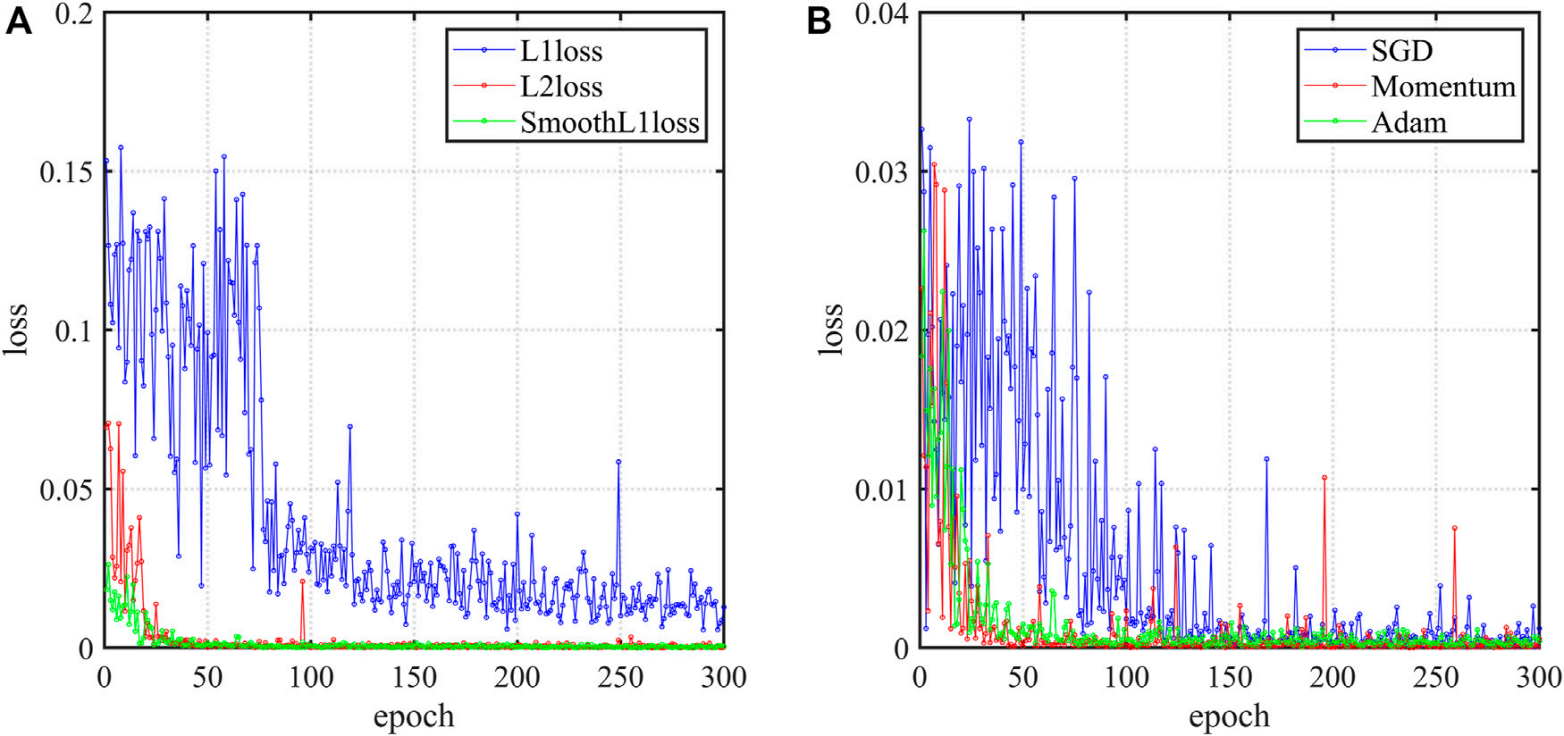
The training process. **(A)** The effect of loss functions on the neural-network; **(B)** The effect of optimizers on the neural-network.

This study is essentially a regression problem. The most commonly used loss functions for regression problems are the mean square error (L2loss) and the mean absolute error (L1loss). L2loss function curve is smooth and can converge quickly to a minimum even at meager learning rates. However, when outliers exist in the training set, L2loss gives higher weight to the outliers, affecting the overall performance (Natekin and Knoll, 2013). L1loss performs sluggishly for outliers but converges slowly. So it is natural to think of the SmoothL1loss function. The SmoothL1loss function converges faster than L1loss. Compared to L2loss, it is insensitive to outliers. To further verify the effect of loss function on the neural-network, Figure 3A shows the training process with three different loss function settings. L1loss has the slowest convergence speed and relatively low training accuracy. In contrast, SmoothL1loss has the fastest convergence speed and the best training accuracy. Therefore, SmoothL1loss is finally chosen as the loss function.

#### 3.2.3 Optimizers

Optimizers can optimize neural-network to improve training accuracy and save training time. The most basic optimizer is the SGD algorithm, initially introduced in the previous subsection. The SGD algorithm is computationally efficient and only requires solving the first-order derivatives of the loss function. However, The SGD algorithm makes the results fall into saddle points and find local optimal solutions because of the direction. Consequently, this paper uses an adaptive optimization algorithm that can update the learning rate automatically. To further verify the effect of the optimizer on the neural-network, Figure 3B shows the training process under the three optimizer settings of SGD, SGD with momentum, and Adam. SGD has the worst optimization effect and the slowest speed. As a modified version of SGD, Momentum is much better. Adam is the best and the fastest convergence speed. So Adam optimizer is chosen.

### 3.3 Generation of the Training Set

The input set is $\left(u_{l},v_{l}\right)$ , $\left(u_{r},v_{r}\right)$ and the output set is $\left(X_{w},Y_{w},Z_{w}\right)$ . A common practice is to use the corner points of the ordinary chessboard as feature points. This method is simple and easy to operate. However, the fisheye camera distortion is severe. The chessboard will be severely distorted at the location closer to the camera, with low or even undetectable corner point detection accuracy at the edge.

**FIGURE 4:**
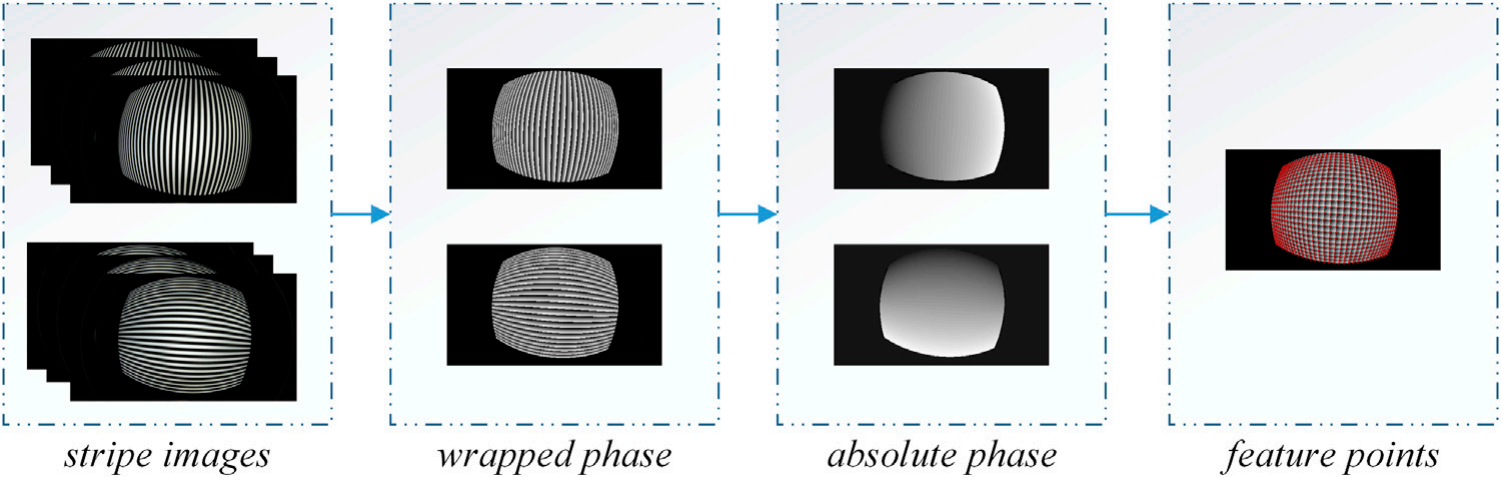
Feature extraction step.

To solve the above problems, the active phase target is used. Firstly, the wrapped phase of the sinusoidal periodic stripe image is solved using the three-step phase shift equation. According to the multi-frequency method, the phase is unwrapped to obtain the absolute phase. Finally, we select the eligible pixel points as feature points according to the absolute phase. A set of exactly matched image coordinates and spatial coordinates will be obtained if the unwrapping is successful. The feature points extracted using our method have the advantage of quantity and are minimally affected by the fisheye camera distortion. Figure 4 shows the specific implementation flow chart, summarized as follows:

1) Generate three-frequency three-step stripe images with equal-step phase shift increments of 2π⁄3. Their intensities can be expressed as: Eq. 11.

$$\begin{array}{c} \{\begin{array}{c} I_{1}=I^{\prime }\left(x,y\right)+I^{\prime \prime }\left(x,y\right)\text{cos}\left[\phi \left(x,y\right)-\frac{2\pi }{3}\right]\\ I_{2}=I^{\prime }\left(x,y\right)+I^{\prime \prime }\left(x,y\right)\text{cos}\left[\phi \left(x,y\right)\right]\\ I_{3}=I^{\prime }\left(x,y\right)+I^{\prime \prime }\left(x,y\right)\text{cos}\left[\phi \left(x,y\right)+\frac{2\pi }{3}\right] \end{array} \end{array}$$ (11)

where $I_{1}$ , $I_{2}$ , and $I_{3}$ are the grayscale values of the phase diagram; $I^{\prime }\left(x,y\right)$ is the background light intensity; $I^{\prime \prime }\left(x,y\right)$ is the intensity modulation parameter; ϕ(x,y) is the wrapped phase to be solved. The horizontal and vertical phase shift stripes are displayed sequentially on the LCD.

2) Two fisheye cameras are fixed on the overhead camera mount, and the LCD monitor is fixed on the high-precision horizontal elevator. The fisheye cameras can shoot the LCD overhead. The high-precision horizontal elevator controls the LCD to move in the $Z_{w}$ direction in steps (the displacement error is 0.05 mm). Two fisheye cameras are controlled in each plane to acquire stripe images simultaneously.
3) According to Eq. 12, a three-step phase shift algorithm is used to calculate the two wrapped phases $\phi_{u}$ and $\phi_{v}$ of the streak image. The value domain of the Arctangent function is within [−π,π] . So if the streak image with more than one period is used for decoding, the calculated wrapped phase is discontinuous. Therefore, the wrapped phases are unwrapped using the multi-frequency method to obtain the continuous absolute phases ${\text{Φ}}_{u}$ and ${\text{Φ}}_{v}$ .

$$\begin{array}{c} \phi \left(u,v\right)={\text{tan}}^{-1}\left(\sqrt{3}\frac{I_{1}-I_{3}}{2I_{2}-I_{1}-I_{3}}\right) \end{array}$$ (12)

4) Any point on the stripe image, calculate its absolute phase ${\text{Φ}}_{u}$ and ${\text{Φ}}_{v}$ . Some alternative feature points can be extracted if they satisfy the following relationships: Eq. 13.

$$\begin{array}{c} \{\begin{array}{c} \left|\Phi_{u}-2\pi n\right|<\tau \\ \left|\Phi_{v}-2\pi m\right|<\tau \end{array} \end{array}$$ (13)

where τ is an artificially set threshold; m and n are integers. Then, among these alternative feature points, the coordinates such that $\left|\Phi_{u}-2\pi n\right|+\left|\Phi_{v}-2\pi m\right|$ obtains the minimum value are searched for as the desired feature points. Finally, least-squares linear interpolation is used to optimize the feature points to the sub-pixel level.

5) The absolute phase is converted to spatial coordinates for each feature point with the following equation: Eq. 14.

$$\begin{array}{c} \left[\begin{array}{c} X_{w}\\ Y_{w} \end{array}\right]=\frac{qP}{2\pi }\left[\begin{array}{c} {\text{Φ}}_{u}\\ {\text{Φ}}_{v} \end{array}\right] \end{array}$$ (14)

where P represents the number of pixels in the stripe cycle; *q* represents the pixel size of the LCD. Use the reading of the high-precision horizontal elevator as the $Z_{w}$ coordinate of the feature point. In this paper, the fisheye camera has a large field of view. The field of view can cover the whole LCD screen even at a position very close to the camera. So the spatial coordinates of the feature points determined by the left and right cameras are the same.

After determining the input and output data sets, the image and spatial coordinates have different value ranges. So it is necessary to normalize the data. Normalization can improve the convergence speed of the neural-network and the model’s accuracy. We use the polar difference transformation method.

## 4 Experiments

**FIGURE 5:**
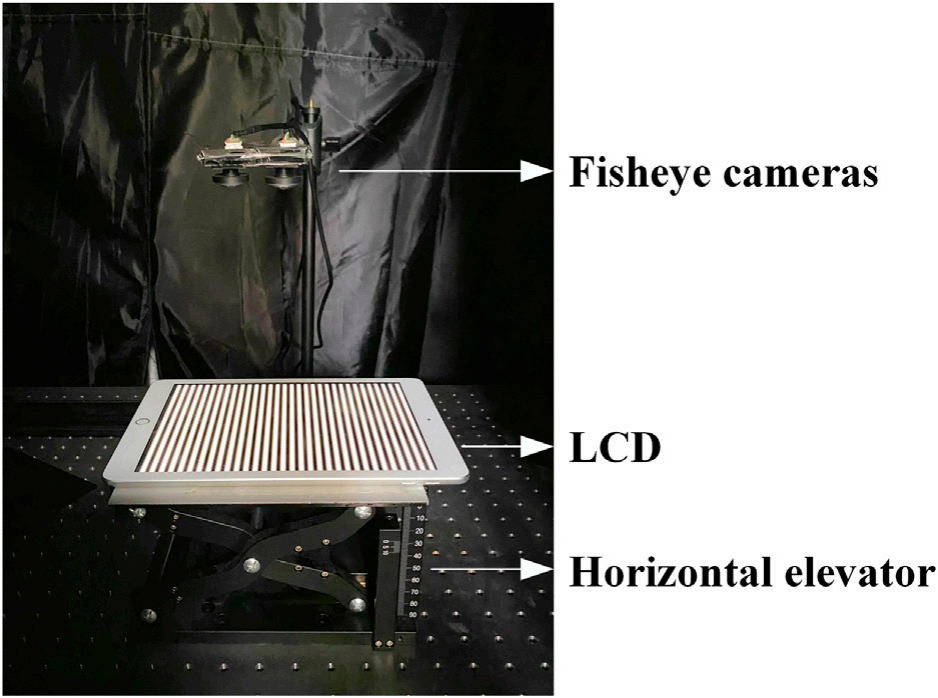
Experiment platform.

To verify the accuracy of the proposed method, an experimental platform was built. Figure 5 shows the experiment platform. The experimental platform includes two identical cameras (AR0230AT), a high-precision horizontal elevator (HTZ210), an LCD (iPad A1893), and a chessboard calibration plate. The fisheye lens (LRCP12014_27 1/2) mounted on the camera has a focal length of 1.4 mm and a field of view of 220°. Two comparison experiments were conducted in different configurations. Finally, the trained neural-network is used to reconstruct the corner points of the chessboard and part of the surface of the sphere.

### 4.1 Neural-Network Method vs. Traditional Fisheye Camera Model Method

The first experiment compares the neural-network-based fisheye camera stereo calibration (the proposed method) with traditional fisheye camera stereo calibration. As shown in Figure 5. Two fisheye cameras are mounted on the overhead camera mount. The LCD is fixed on a high-precision horizontal elevator. The LCD is used to display the three-frequency, three-step stripe images. The high-frequency stripe period is 64, and the high, medium, and low frequencies multiplier is 6. The LCD resolution is 2048 × 1536 pixels, and the pixel size is 0.096 mm. The high-precision horizontal elevator controls the gradual movement of the LCD in the $Z_{w}$ direction.

The training set is obtained by following the steps described in Section 3.3. The neural-network is configured according to Section 3.2.

**FIGURE 6:**
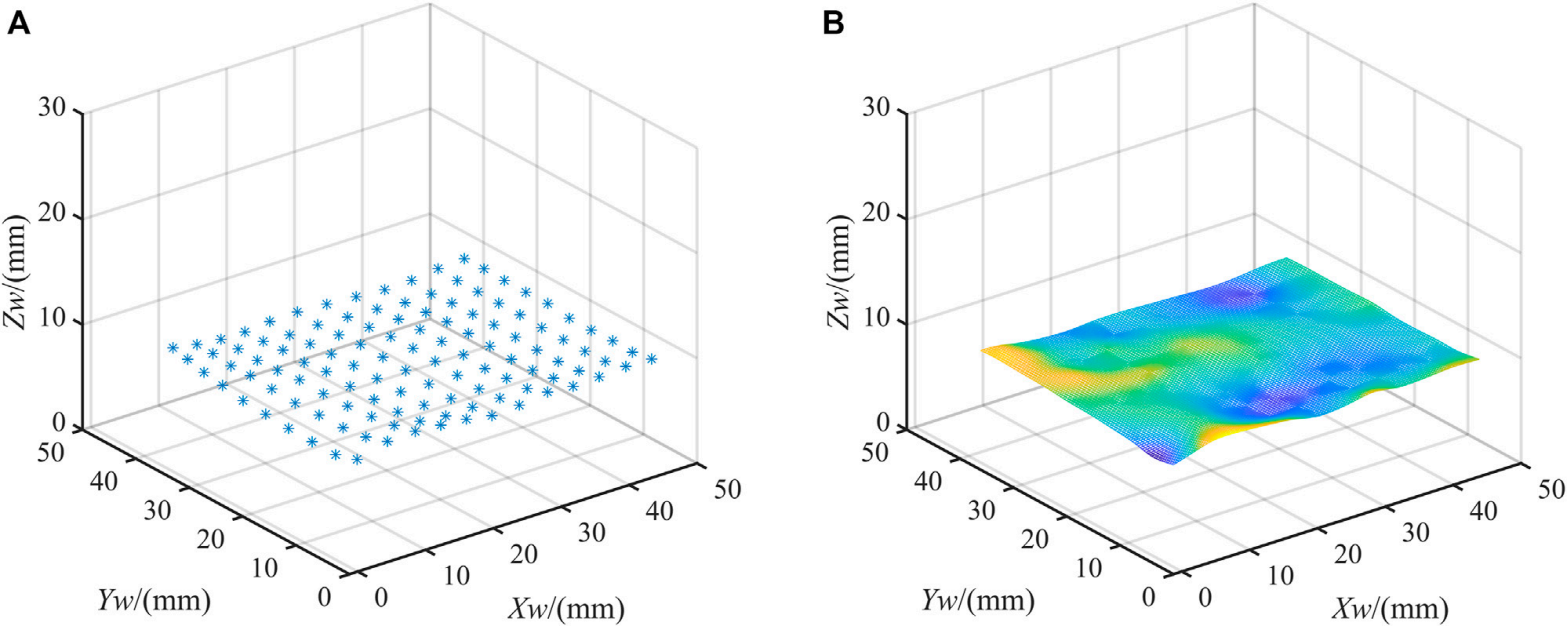
Results. **(A)** Prediction results of sample points; **(B)** The plane fitting results.

Based on the trained network, the sample data can be predicted. Figure 6A shows the prediction results of 120 sample points. The actual values of the spatial coordinates are known. So we can quantitatively analyze the deviations in three directions. The mean error of $X_{w}$ is 0.416 mm, $Y_{w}$ is 0.253 mm, and $Z_{w}$ is 0.271 mm. To visually show the prediction results, the predicted results of the spatial coordinates of the sample points are linearly interpolated. Figure 6B shows the fitted plane.

**FIGURE 7:**
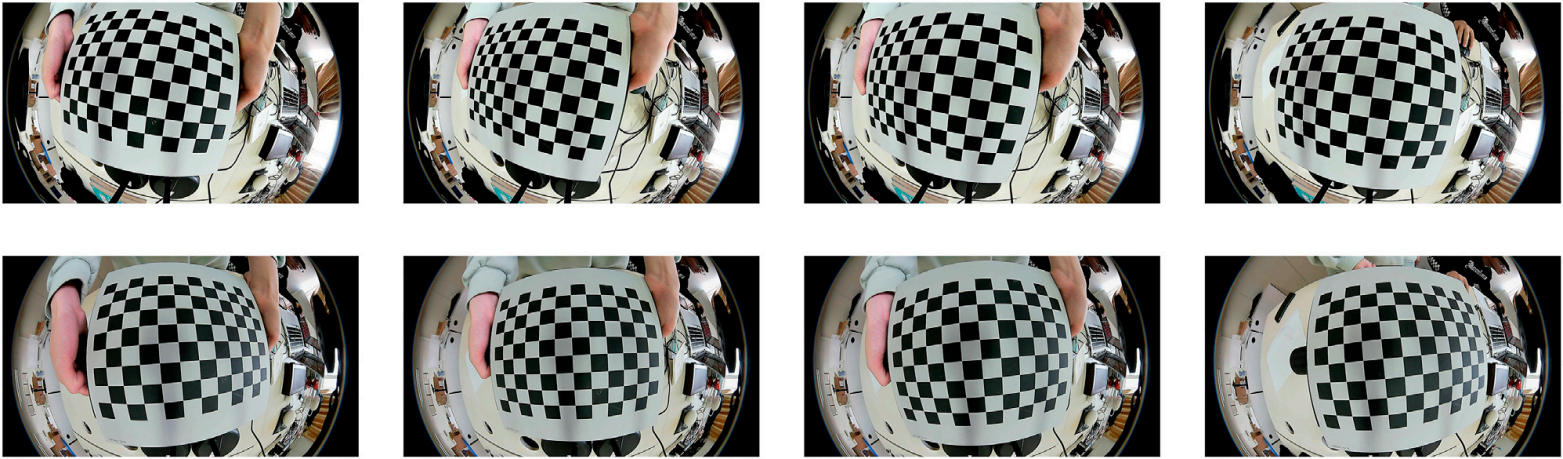
Eight images for conventional stereo calibration. The top four are taken by the left camera; the bottom four are taken by the right camera.

We perform the traditional fisheye camera stereo calibration using the fisheye camera calibration method in opencv3.0. The specific principle can be referred to (Kannala and Brandt, 2006) and is not described in detail here. This method requires two fisheye cameras to take pictures of the target in different directions. A total of 25 images were taken. Figure 7 shows some of the 25 images.

Table 1 compares the reconstruction accuracy of the neural-network model-based method with the traditional fisheye camera model method. The experimental results show that the neural-network-based method proposed in this paper has higher accuracy and is more suitable for fisheye camera with larger distortion.

**TABLE 1:** Mean errors in $Z_{w}$ .

Stereo calibration methods	Mean error in (mm) $Z_{w}$
Neural-network model	0.271
Fisheye camera model	3.967

### 4.2 Active Phase Target vs. Chessboard

The second experiment compares two different methods of obtaining the training set for the neural-network. One is to use active phase targets as proposed in this paper, and the other is to use a chessboard as the target. The experimental procedure using the active phase target has been described in Section 4.1.

The experimental chessboard contains 88 corner points with a spacing of 15 mm and a manufacturing error of 0.01 mm. The Harris corner point detection algorithm can obtain the sub-pixel image coordinates of the chessboard corner points. The corner points of the chessboard are used as feature points. To ensure the consistency of the experimental conditions, the positions of the fisheye cameras are not changed. The chessboard is fixed on the high-precision horizontal elevator. The high-precision horizontal elevator controls the chessboard to move in the $Z_{w}$ direction in steps. The readings of the high-precision horizontal elevator are used as the $Z_{w}$ coordinates of the feature points. The neural-network parameter settings are not changed.

**FIGURE 8:**
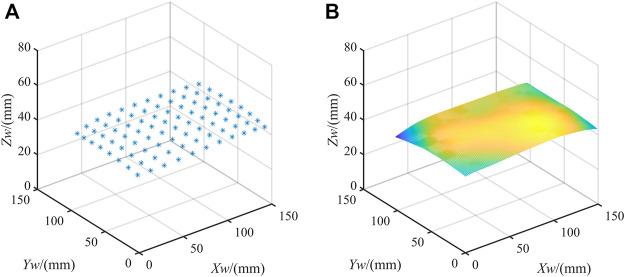
Results. **(A)** Prediction results of sample points; **(B)** The plane fitting results.

The sample data are then predicted based on the trained network model. Figure 8A shows the results. The actual values of the spatial coordinates of these points are known. So we can quantitatively analyze the deviation in three directions. The mean error of $X_{w}$ is 1.105 mm, $Y_{w}$ is 0.894 mm, and $Z_{w}$ is 1.177 mm. To demonstrate the experimental results more intuitively, linear interpolation is performed on the predicted results. Figure 8B shows the fitted plane.

**FIGURE 9:**
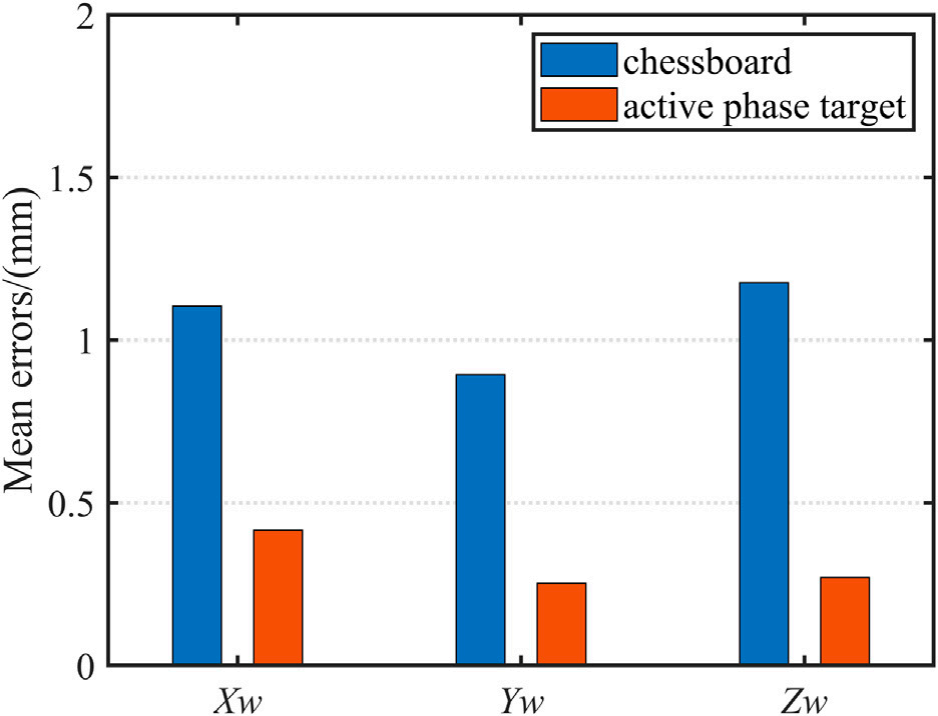
Mean error comparison histogram.

Table 2 compares the reconstruction accuracy comparison of the training set obtained using the active phase target and the chessboard. Figure 9 shows the mean error comparison graph. It is clear that the method using the active phase target to extract feature points as the training set is more accurate, especially in the $Z_{w}$ direction. The experimental results prove that the active phase target has the advantage of the number of feature points and is more suitable for the calibration of the fisheye camera.

**TABLE 2:** Mean errors in $X_{w}$ , $Y_{w}$ , and $Z_{w}$ .

Stereo calibration methods	Mean error in (mm) $X_{w}$	Mean error in (mm) $Y_{w}$	Mean error in (mm) $Z_{w}$
Active phase targets	0.416	0.253	0.271
Chessboard	1.105	0.894	1.177

### 4.3 3D Reconstruction

To further verify the practicability of the proposed method, 3D reconstructions of the chessboard corners and a partial plane of the sphere are performed.

**FIGURE 10:**
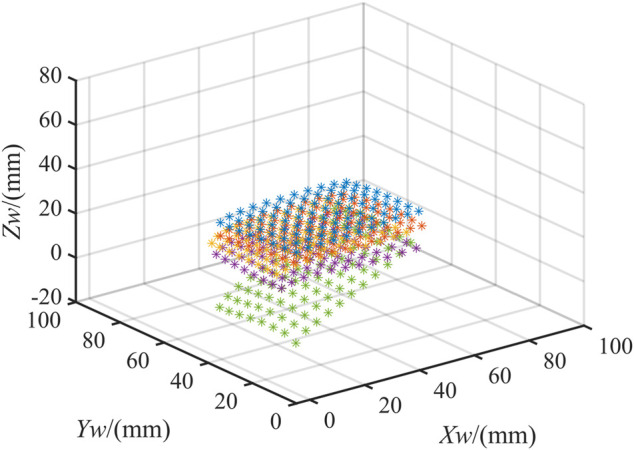
Chessboard cornors reconstruction results.

The experimental chessboard contains 88 corner points with a spacing of 4.9 mm. The binocular fisheye camera takes pictures of the chessboard in different poses at the same time. The subpixel image coordinates of the chessboard corners are obtained using the Harris corner detection algorithm. The spatial coordinates of these corners are then reconstructed using the trained neural-network model. Figure 10 shows the reconstruction results. We calculate the square size of the chessboard based on the spatial coordinates and compare it with the true value. Among them, the reconstruction error of the corners located at the edge of the chessrboard is larger, and the reconstruction errors of the middle corners is smaller. This is due to the characteristics of the fisheye image itself. The edges of the fisheye image are stretched due to the severe distortion of the camera. Table 3 shows the mean error for the chessboard square size.

**TABLE 3:** Mean errors in square size.

Pose	1 (mm)	2 (mm)	3 (mm)	4 (mm)	5 (mm)
Mean errors	0.174	0.066	0.159	0.142	0.345

**FIGURE 11:**
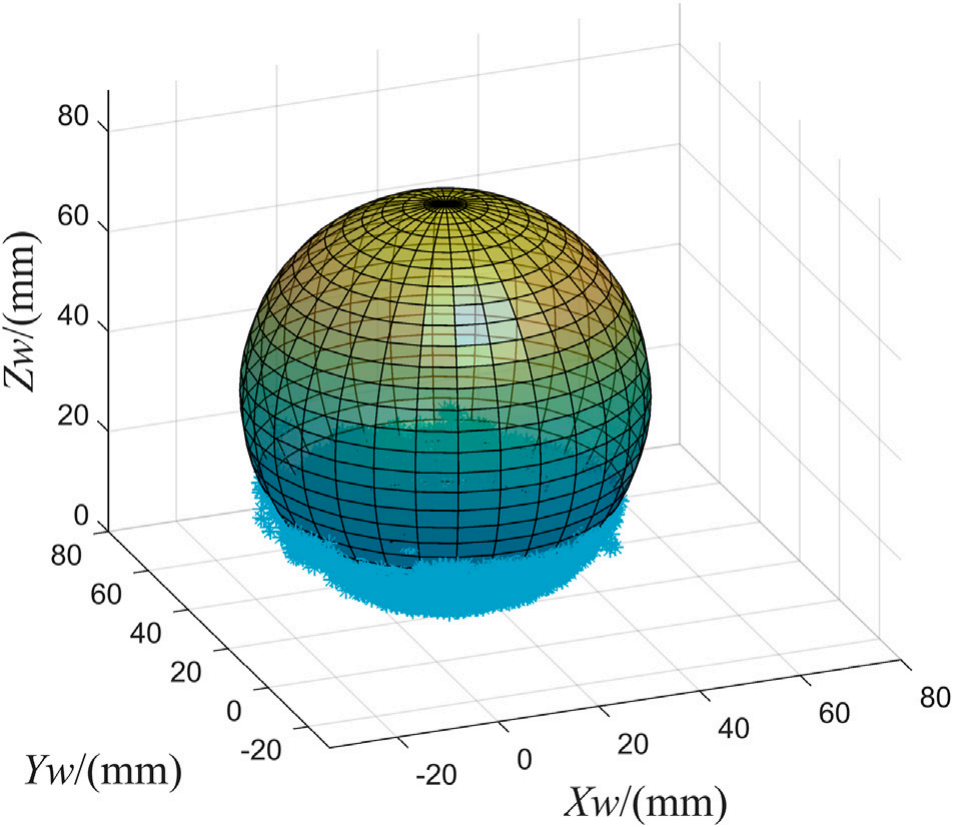
Sphere reconstruction result.

Similarly, we reconstructed a partial plane of the sphere. We recover the absolute phase of the sphere by projecting a fringe image on the upper surface of the sphere. According to the matching relationship between the absolute phases of the sphere in the left and right cameras, the pixel points are matched (Chen X. et al., 2021). Then we use the trained neural-network to predict the spatial coordinates of these points. Figure 11 shows the reconstruction results. We performed a least squares fit to the results for the sphere. The real diameter of the sphere is 71 mm. The fitted diameter is 73.7288 mm. So the reconstruction error is 2.7288 mm. Experiments show that the neural-network-based method proposed in this paper has high measurement accuracy.

## 5 Conclusion

This paper applies a neural-network to the fisheye camera stereo calibration technique. There is no need to pre-build the fisheye camera model. The proposed method is model-free. A nonlinear mapping relationship between image coordinates and spatial coordinates is established using neural-network. The use of the active phase target enables the extraction of feature points with a larger number and higher precision, which is more suitable for the calibration of fisheye cameras. Due to the flexible structure of the neural-network, the neural-network model can be easily extended to the joint calibration of multiple fisheye cameras and the joint calibration of asymmetric fisheye camera layouts. These are expected to be further investigated and implemented in the future.

## Data Availability

The raw data supporting the conclusion of this article will be made available by the authors, without undue reservation.
